# Biology, technology and a bit of serendipity: an interview with Shiva Malek

**DOI:** 10.1242/dmm.049214

**Published:** 2021-09-09

**Authors:** Shiva Malek


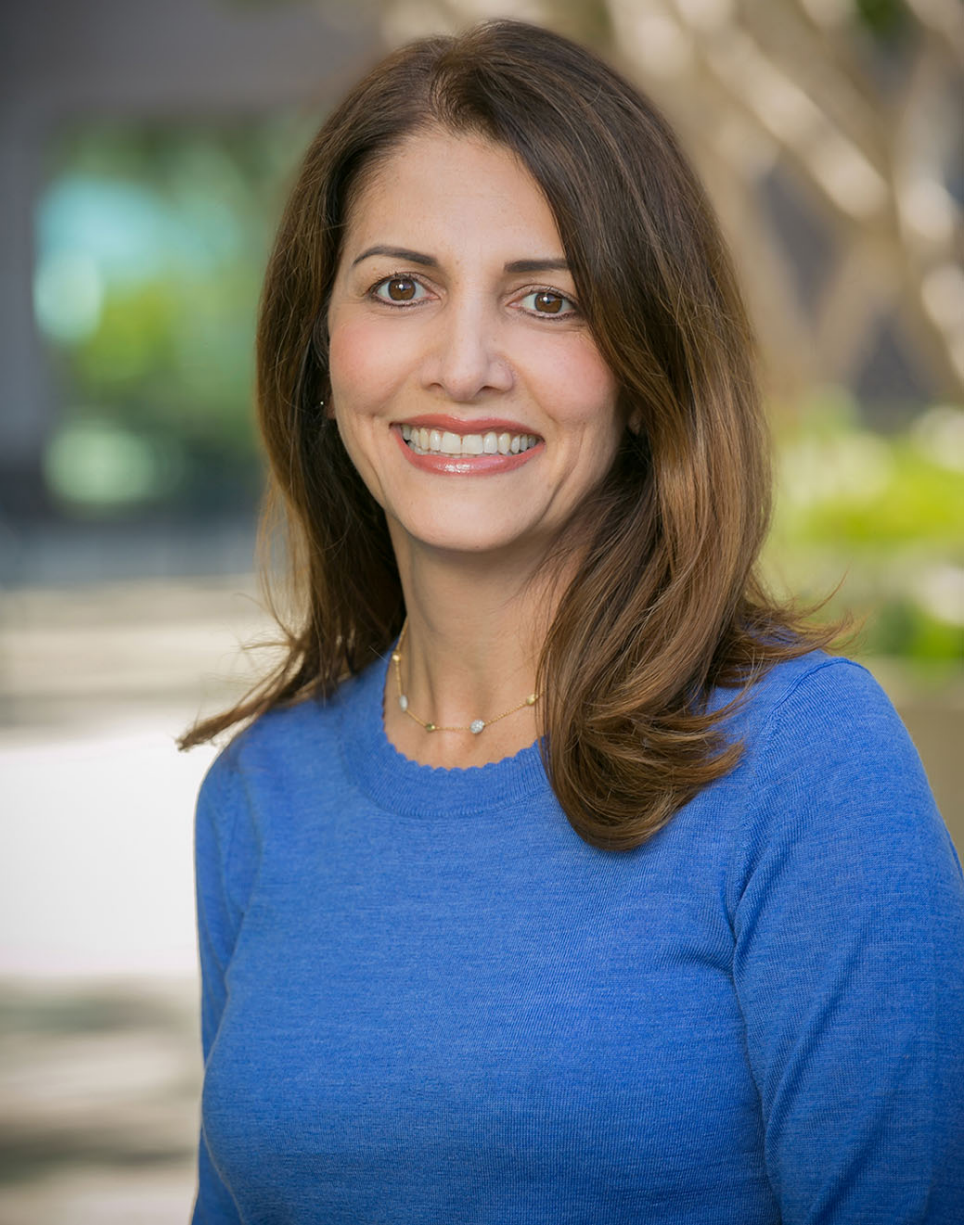


Dr Shiva Malek is Vice President of Discovery Oncology at Genentech, where she oversees the identification of oncology therapeutic targets and drug development. Dr Malek obtained her PhD in biochemistry from the University of California San Diego (UCSD). Post-PhD, she embarked on a scientific career in industry, first in San Diego and then in the Bay area. She joined Genentech in 2006 as one of the first scientists in their newly established small-molecule discovery team. Her work has been instrumental in the discovery and validation of drug targets in various kinase signalling pathways in cancer, paving the way for successful translation of fundamental concepts in cancer biology to effective therapies for patients. She is also a passionate mentor to young scientists and a role model to many. In this interview, Shiva talks about her conventional-wisdom-defying career path, drugging the undruggable and the joys of discovering San Francisco's natural beauty.

**Did you always want to be a scientist?**

I started out wanting to be a physician. We moved to the USA from Iran when I was 7 years old. I didn't speak much English, so science and math were easier for me, and, being an immigrant, my parents encouraged me to either be an engineer or a doctor. So I never considered anything other than being a doctor. It wasn't until my freshman year in college at University of California, Los Angeles (UCLA), when I joined the lab of Dr Juli Feigon [currently Distinguished Professor at UCLA's Department of Chemistry and Biochemistry], that I realised I had a passion for basic science. I worked with her from the summer after freshman year until I graduated and this was a turning point for me.

**It's interesting that you were part of a lab as an undergrad for such a long time**

It was a super interesting experience. UCLA is a big school and it's hard to get to know any of your professors when you are in a 300-plus class. Like any good pre-med major, I realised I needed to do research. I first approached Dr Feigon as a pretty clueless 17-year-old freshman, thinking I'll get a project in her lab. She responded that she actually needed a dishwasher. This humbled me a bit, but was a great learning experience. I realised she was the first non-tenured faculty to be hired at UCLA and later became the first woman to get tenure at the Department of Chemistry and Biochemistry. She has this fire in her belly and is a phenomenal nuclear magnetic resonance (NMR) spectroscopist, but she pushed me really hard. Back then, I was too young to understand the pressure or even the gender politics. Looking back, I appreciate she was and still is a force of nature and she had to be tough to persist in that environment. I gradually moved on to making buffers and preparing DNA samples and finally got my own project and my name on papers. It was amazing to learn how to do research in a lab and to learn from a woman who, at the time, was still building her career.

**You transitioned to the industry fairly early in your career. What motivated you to transition?**

This was not entirely intentional. I finished my PhD in 2000 during the biotech boom. You could barely finish defending your thesis before people would try to recruit you. And this was in San Diego, which was not a major hub like San Francisco, Cambridge or Boston. I was interviewing for academic postdoc positions, but also had other job offers. I ended up taking a position at Aurora Biosciences, which was co-founded by Charles Zuker, Michael Geoffrey and the late Roger Tsien, a Nobel laureate. Roger was on my PhD committee, so I knew him from UCSD, and he was heavily involved in the company as well as the University. What I liked was the company's idea of using biophysical methodologies that Roger pioneered to understand protein function in cells and develop screens for therapies. There was a bioengineering component that I really liked. Having come from a structural biology and biochemistry background in Gouri's lab [Dr Gourisankar Ghosh was Shiva's PhD advisor at the Department of Chemistry and Biochemistry] at UCSD, I really wanted to take those structural and functional insights and study them in cells. I thought the job at Aurora would give me that opportunity. The company's platform was essential for Vertex Pharmaceuticals’ ground-breaking advances for treatment of cystic fibrosis. I learned a ton through this experience, and I knew I wanted to do more translational work and apply my science in a way that would benefit patients.

If I were to do things again, I honestly do not know if I would go straight into industry. At the time, many of my advisors thought it was somewhat taboo to go into industry and told me I was making a mistake. Things turned out great, but now, mentoring postdocs, I think the period of time after a PhD is important for learning independence and exploring something different. At the end of the day, it boiled down to what I wanted to do. So, although it's great to take advice, ultimately, there is no one career path – you have to do what feels right for you, even if that means going against that advice!

**Belinda Cowling recently wrote an article for us (Cowling and Thielemans, 2020), in which she discussed how the vast majority of candidate drugs for rare neuromuscular disorders get lost in the bench-to-clinic pipeline, regardless of whether the bench discoveries originate in academia or in industry. Can you tell us how this looks in oncology?**

In terms of therapies, oncology has been a trailblazer for using (cancer) genomics to understand the underlying causes, particularly oncogenes, and using this to develop therapies. Unlike with many other diseases, cancer has more space for functional genomics and for drugging the drivers of disease. In the past few decades, the paradigm has been ‘see mutation, drug mutation’, which brought us the many clinically successful kinase inhibitors. Now we have pan-tumour approvals for targeting the driver mutation regardless of the cancer type. We can do that because cancer patients get sequenced as part of their diagnosis. The challenge, and what I think is the biggest existing gap in oncology, is that we cannot drug every mutation. We can understand the function, but either the oncogene is not easy or safe to drug or the disease is driven by loss of a tumour suppressor. However, technological advances will help with these challenges. It's not enough to understand the biology, you also need the right technology to target the dependency. And this is a very interdisciplinary problem that we can solve by integrating expertise from many fields.

“It's not enough to understand the biology, you also need the right technology to target the dependency. And this is a very interdisciplinary problem that we can solve by integrating expertise from many fields.”

The technological advances and discoveries in academia have played a huge role in both understanding the biology and how to target some of the hard-to-drug drivers. Kevan Shokat made seminal progress in targeting KRAS^G12C^ in 2013 and opened the door to drugging KRAS. I imagine drugging other non-druggable targets like transcription factors will be the next era. We still have a lot of work to do, but the human genetic data are phenomenal, and as we learn more and identify more drivers, we will be able to safely drug those causal variants.

**You mentioned KRAS, one of the most frequently mutated oncogenes in cancer. It's a transmembrane protein with enzymatic activity – on paper, a good therapeutic target. What makes KRAS so hard to drug?**

Conventional wisdom about KRAS’ enzymatic activity…it's a GTPase, tells you that all you have to do is inhibit this function like you would do with any other enzymatic target. But the challenge is that mutations typically trap the enzyme in a GTP-bound state. Its affinity for GTP is in the picomolar range, but there are millimolar levels of GTP in the cell, so GTP-competitive inhibitors are not an option. Additionally, despite decades of people looking, there are no other druggable pockets. Overcoming this challenge took a few things, as we discuss in a recent Perspective (Rudolph et al., 2021): technological breakthroughs; for example, Kevan developed an innovative tethering screening approach for KRAS^G12C^; a bit of serendipity, which is always welcome in science; and the confidence to challenge the dogma. So it's not enough to understand the underlying biology, successful inhibition of KRAS and other GTPases also requires the right screening technology.

**It took 40 years from Varmus and Bishop's discovery of RAS to Kevan Shokat's first successful inhibitor, but there are currently a number of KRAS^G12C^ inhibitors in clinical trials**

That's correct. There are many KRAS^G12C^ inhibitors in the clinic, and the more options patients have, the better off they will be. We can also learn how different tumours respond to different inhibitors, and we can develop combinations. And the quicker we can get drugs to patients, the better off patients are. Companies are perceived to compete with one another, but, for us, another company is not the enemy, cancer is the enemy. If someone else successfully targets KRAS, I am grateful that the field is able to make a difference in patients’ lives. And I hope that Genentech will be able to contribute.

“Companies are perceived to compete with one another, but, for us, another company is not the enemy, cancer is the enemy.”

**Switching from RAS to RAF. BRAF inhibitors have been a game changer in melanoma, but resistance became a problem. Do you think RAS inhibitors may be subjected to the same quick resistance emergence?**

I don't have a crystal ball, but there are a lot of parallel lessons between the BRAF story and KRAS. Like with BRAF, we see both intrinsic and lineage-dependent resistance emerging with KRAS inhibitors. We see great responses in KRAS-driven lung cancer, but hardly any in colorectal cancer, which mirrors the BRAF-driven melanoma versus colorectal cancer differences. We are exploring combinations that may give us more durable responses or responses in cancer types, like colorectal, that quickly undergo adaptive reprogramming to escape the drug. Those are key questions that will need to be addressed clinically once we have more data from KRAS inhibitor trials, but we can apply the lessons learned in the BRAF field. A lot of labs, including mine, are modelling resistance preclinically, but until we see data from bigger clinical trials, it's impossible to predict how this plays out in cancer patients. I'm expecting to see some surprising mechanisms.

Of course, another area that Genentech and other companies are looking into is treatment of early-stage disease. That's when a cancer is less heterogeneous, and the thinking is that intervening with targeted therapies earlier may result in more durable responses. A driver mutation, if it is indeed the driver, should be present in early-stage disease as well, so your target is there. Anecdotally, we know that targeted therapies are very effective in paediatric patients, which tend to have genetically less complex tumours.

**Switching gears a bit. You are recognised as an incredibly supportive mentor, but there is so much nuance in mentor-mentee relationships. Is there a ‘secret sauce’ to good mentorship?**

Good question. I do think that the best mentor-mentee relationships are not forced; there is a friendship and an element of give and take, and both learn from each other. As a mentor, I learn a ton from my trainees and that is part of that special sauce. I would encourage people, both those in mentee and those in mentor positions, to listen to each other and learn from each other.

“[…] the best mentor-mentee relationships are not forced; there is a friendship and both learn from each other. As a mentor, I learn a ton from my trainees and that is part of that special sauce.”

I intentionally hire postdocs from different backgrounds, both scientific and otherwise. Part of how we all grow is learning from each other. By mentoring, I learn how to better leverage my leadership skills and how to approach a scientific question differently. It's never one-sided.

**As a woman in a leadership role, and an immigrant, are there any measures to support women and other under-represented groups that you are particularly mindful of?**

Absolutely. I am on the Diversity and Inclusion board at Genentech and a member of the AACR Women in Cancer Research Council. I also get so much joy out of the personal connections and relationships as a mentor of trainees in my lab. I have routinely recruited young women scientists as interns in my lab since I started at Genentech. Coming from a Middle-Eastern background, I naturally connect with people from that area, both refugee and immigrant scientists. I am drawn to hearing their stories and am interested in their approach to research. I'm mindful about whom I can support, either by an internship or postdoc position in my lab or by recommending them for positions within my department. We are all richer by their presence.

As I got to a more senior level, implementing positive change has become easier as I now have a platform. I take this responsibility very seriously, and I empower junior colleagues to push for further change. We are very fortunate to have a number of women in senior positions at Genentech and we work incredibly well together, so I'm usually not the only voice in the room. A single woman may not always feel comfortable speaking up, and there is still much that needs to be done. I'd like to see more women give keynote addresses at major international conferences. And it would be nice if one day we didn't have to consciously think about gender parity and diversity.

**And finally: what do you enjoy doing outside of the lab?**

I have two teenage sons and a dog, and the thing I enjoy the most is going hiking with them. I love being outdoors. There is this great spot here in San Francisco called Fort Funston; it's right by the beach.
